# Early Advanced Airway Management and Clinical Outcomes in Out-of-Hospital Cardiac Arrest: A Nationwide Observational Study

**DOI:** 10.3390/jcm14217652

**Published:** 2025-10-28

**Authors:** Jung Ho Lee, Dahae Lee, Eujene Jung, Hyun Ho Ryu, Jeong Ho Park, Young Sun Ro, Kyoung Jun Song

**Affiliations:** 1 Department of Emergency Medicine, Chonnam National University Hospital, Gwangju 61469, Republic of Korea; sw97220501@naver.com (J.H.L.); dhlee115@naver.com (D.L.); 2 Laboratory of Emergency Medical Services, Seoul National University Hospital Biomedical Research Institute, Seoul 03080, Republic of Korea; timthe@gmail.com (J.H.P.); ro.youngsun@gmail.com (Y.S.R.); 3 Department of Emergency Medicine, Chonnam National University Medical School and Hospital, Gwangju 61469, Republic of Korea; 4 Disaster Medicine Research Center, Seoul National University Medical Research Center, Seoul 03080, Republic of Korea; 5 Department of Emergency Medicine, Seoul National University College of Medicine and Seoul National University Boramae Medical Center, Seoul 03080, Republic of Korea; skciva@gmail.com

**Keywords:** out-of-hospital cardiac arrest, advanced airway management, emergency medical services, propensity score matching, structural equation modeling, neurological outcomes

## Abstract

**Background/Objectives**: Out-of-hospital cardiac arrest (OHCA) has persistently low survival rates. While advanced airway management (AAM) is crucial during cardiopulmonary resuscitation, optimal timing remains unclear. This study examined the association between early AAM and clinical outcomes in adult OHCA patients. **Methods**: This retrospective study analyzed Korean nationwide OHCA registry data (August 2019–December 2022). Adult patients with emergency medical service-treated OHCA of presumed medical origin receiving AAM were included. Early AAM was defined as airway placement within 5 min of CPR initiation. Time-dependent propensity score matching controlled for selection bias and time-related confounding. Structural equation modeling examined associations between AAM timing and other prehospital interventions. Primary outcome was survival to hospital discharge with good neurological recovery (cerebral performance category 1–2). **Results**: Among 51,869 patients receiving AAM, 27,591 received early AAM and 24,278 received delayed AAM. After propensity score matching, 12,014 patients were included per group with balanced characteristics. Early AAM was associated with higher prehospital return of spontaneous circulation (11.8% vs. 10.5%; adjusted RR 1.21, 95% CI 1.12–1.29) and favorable neurological recovery (5.8% vs. 5.1%; adjusted RR 1.12, 95% CI 1.01–1.23). AAM timing correlated with timing of other critical interventions, including rhythm analysis and epinephrine administration. **Conclusions**: Early AAM within 5 min of CPR initiation was associated with improved neurological outcomes and increased prehospital ROSC in OHCA. Airway timing may indicate overall resuscitation quality, emphasizing the importance of coordinated, timely prehospital interventions.

## 1. Introduction

Out-of-hospital cardiac arrest (OHCA) remains a major global public health challenge, with survival rates ranging from 3% to 20% and a substantial proportion of survivors experiencing severe neurological impairment [1]. Despite advances in emergency medical services (EMS), overall survival remains low [1,2]. Because critical determinants of survival and neurological recovery are often established in the prehospital phase, recent attention has focused on early EMS interventions [1]. There is increasing interest in identifying prehospital strategies that not only improve survival but also enhance neurological outcomes and long-term quality of life [3].

Among prehospital interventions, advanced airway management (AAM) is essential in cardiopulmonary resuscitation (CPR) for securing the airway and ensuring adequate ventilation [4]. AAM includes procedures such as tracheal intubation and supraglottic airway insertion, performed during OHCA by trained EMS personnel. Guidelines from organizations including the American Heart Association and European Resuscitation Council endorse the use of tracheal intubation or supraglottic airways, while acknowledging that the optimal airway strategy and timing remain subjects of ongoing investigation [5,6].

Although several studies have investigated the impact of AAM on patient outcomes, results have been inconsistent, particularly regarding intervention timing [7]. Some observational studies report that early AAM is associated with higher rates of return of spontaneous circulation (ROSC) and more favorable neurological outcomes [8,9], whereas others suggest possible harm due to interruptions in chest compressions and delays in other time-sensitive interventions [10,11]. These discrepancies may reflect differences in EMS system characteristics, including training, protocols, and types of airway devices used [7]. This underscores the need for further research across diverse EMS settings to clarify the effects of airway timing on outcomes.

This study aimed to investigate the association between early AAM and clinical outcomes in adult OHCA patients while minimizing resuscitation time bias. Using a nationwide EMS dataset from Korea, where supraglottic airway devices such as i-gel accounted for over 90% of advanced airway placements, we applied time-dependent propensity score matching (TDPSM) to compare early and delayed AAM groups. Additionally, structural equation modeling (SEM) was employed to examine how AAM timing is influenced by, and interacts with, other prehospital interventions such as defibrillation and epinephrine administration. This approach offers a comprehensive perspective on the temporal coordination of multiple procedures during prehospital resuscitation and clarifies the role of AAM within this process.

## 2. Materials and Methods

### 2.1. Study Design and Setting

This retrospective observational study used data from the nationwide OHCA registry in South Korea, maintained by the National Fire Agency (NFA). The registry provides comprehensive information on prehospital interventions and in-hospital outcomes.

The Korean EMS system is a publicly operated, fire-based system managed by the NFA, comprising 18 provincial fire departments and dispatch centers. A national emergency number (119) is used for EMS, fire, and rescue services. All EMS providers are trained in basic life support, while advanced emergency medical technicians and nurses are authorized to perform advanced life support, including AAM, intravenous (IV) access, and medication administration under online medical direction. Approximately 63% of EMS responses involved three personnel, and 79% of OHCA cases received multi-tier dispatch. Most ambulance teams (97%) included at least one advanced emergency medical technician or nurse. According to NFA protocols, EMS providers must perform at least 5 min of on-scene resuscitation, and all OHCA patients are transported to the hospital unless ROSC is achieved [12].

Post-resuscitation care is delivered in emergency departments classified into three levels by the Ministry of Health, based on staffing and resources. Level 1 and 2 centers have 24/7 emergency physicians, while Level 3 centers may rely on general physicians. National guidelines, based on AHA recommendations, standardize post-resuscitation care.

This study was approved by the Institutional Review Board of Seoul National University Hospital (IRB No. SNUH-1103-153-357, 30 March 2024). The requirement for informed consent was waived due to the retrospective nature of the study.

### 2.2. Data Sources

Data were obtained from the nationwide OHCA registry maintained by the NFA of Korea. The registry integrates data from multiple sources, including EMS run sheets, dispatcher-assisted CPR records, the EMS cardiac arrest registry, and hospital medical records.

Hospital-based interventions and outcomes were collected by trained medical record review professionals affiliated with the Korea Disease Control and Prevention Agency. These certified reviewers extracted data according to nationally standardized protocols based on the Utstein template [13]. To ensure data quality and validity, a multidisciplinary quality control committee—including emergency physicians, epidemiologists, statisticians, and data reviewers—conducted regular audits and provided monthly feedback to reviewers [14].

### 2.3. Study Population

This study included adult patients (aged ≥18 years) who experienced EMS-treated OHCA of presumed medical etiology between August 2019 and December 2022. The study period was determined by data availability, as the Korean nationwide OHCA registry began systematically collecting time-stamped data for advanced airway management in August 2019. Among 107,987 OHCA cases recorded in the nationwide EMS registry, patients were excluded if they were under 18 years of age, had a non-medical cause of arrest, did not receive AAM, or had missing information on the timing of CPR initiation or AAM. Patients who achieved ROSC prior to or at the time of advanced airway placement were also excluded, as the objective was to evaluate the association between airway timing and clinical outcomes before ROSC.

### 2.4. Variables and Measurements

The primary exposure variable was the timing of prehospital AAM, measured as the interval in minutes from EMS-initiated chest compressions to airway device placement. In addition to airway timing, other key prehospital interventions were recorded and analyzed based on their intervals from CPR initiation. These included time to first rhythm analysis using an automated external defibrillator, IV line insertion, epinephrine administration, and ROSC.

All time-stamped variables were recorded by EMS personnel at the scene using standardized run sheets. Timing was based on direct observation, synchronized on-scene clocks, or retrospective review of dispatch and device data. To ensure consistency, all intervention times were calculated as minute-based intervals from the start of EMS-initiated CPR.

Additional variables included patient demographics, comorbidities, arrest characteristics, and EMS operational. In-hospital management data included ROSC at emergency department arrival, targeted temperature management (TTM), percutaneous coronary intervention, extracorporeal membrane oxygenation (ECMO), and neurological outcome at discharge.

### 2.5. Outcomes

The primary outcome was survival to hospital discharge with good neurological recovery, defined as a cerebral performance category score of 1 or 2 [15]. Secondary outcomes included survival to hospital discharge and prehospital ROSC.

### 2.6. Statistical Analysis

This study assessed the association between early AAM and clinical outcomes among adult OHCA patients. The timing of AAM was measured from the start of CPR performed by EMS providers, with early AAM defined as advanced airway placement within 5 min of CPR initiation. This cutoff was based on the median airway time, ensuring a balanced distribution between early and delayed groups.

To minimize selection bias and address time-dependent confounding, we employed TDPSM [16]. The propensity score was estimated using a Cox proportional hazards model, with the timing of AAM as the dependent variable. Time zero was defined as the initiation of CPR by EMS providers, as patients became at risk for AAM from that point forward.

Propensity scores incorporated a comprehensive set of covariates: age, sex, medical history, urbanization level of arrest, witnessed status, location of arrest (public or private), bystander CPR, initial shockable rhythm, EMS response type (multi-tier response), EMS operational times (response, scene, and transport times), Emergency department level, type of advanced airway device, and whether IV line insertion or prehospital epinephrine was performed. Intervention timing variables—such as time to epinephrine administration, IV line insertion, and defibrillation—were excluded from the matching model because these procedures were not universally performed. Since timing variables are defined only for patients who received the intervention, including them would not necessarily improve covariate balance. We conducted 1:1 nearest-neighbor matching without replacement within time-dependent risk sets [16,17], with exact matching on year of arrest to control for secular trends. The caliper width for matching was set at 0.2 standard deviations of the logit of the propensity score. Covariate balance was evaluated using standardized mean differences (SMDs), with SMD < 0.25 indicating adequate balance [18].

We used a generalized estimating equation model with a log link to estimate risk ratios (RRs) and 95% confidence intervals (CIs) for prehospital ROSC, survival to hospital discharge, and favorable neurological recovery. Both unadjusted and adjusted models were reported. The adjusted generalized estimating equation model included variables such as age, sex, medical history, urbanization level, public arrest location, bystander CPR, multi-tier EMS response, response time, type of advanced airway, and scene time.

To further investigate how AAM timing may be affected by other EMS interventions and system factors, we performed SEM [19]. SEM is a statistical technique that enables simultaneous estimation of multiple interrelated pathways, accounting for measurement error. This approach is particularly suited to modeling complex, time-dependent clinical processes such as prehospital resuscitation. In this study, SEM was used to assess relationships between airway delay and other time-sensitive prehospital procedures, providing a comprehensive perspective on airway placement within the sequence of resuscitative actions.

All statistical analyses were conducted using R version 4.3.0 (R Foundation for Statistical Computing, Vienna, Austria). SEM was performed using the lavaan package. Statistical significance was defined as a *p*-value < 0.05.

## 3. Results

### 3.1. Patients

**Figure 1:**
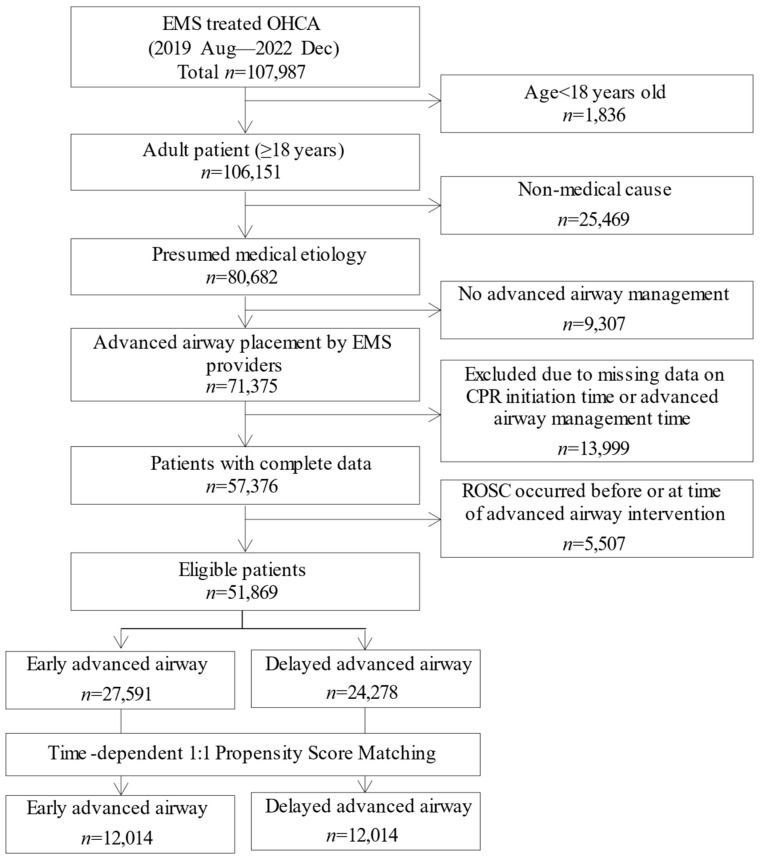
Patient flow chart. EMS—emergency medical services, OHCA—out-of-hospital cardiac arrest, ROSC—return of spontaneous circulation, CPR—cardiopulmonary resuscitation.

Between August 2019 and December 2022, 107,987 patients with EMS-treated OHCA were identified. After excluding 1836 patients < 18 years, 106,151 adults remained. Of these, 80,682 had a presumed medical cause of arrest and were eligible for further analysis. We then excluded 9307 patients who did not receive AAM, resulting in 71,375 who underwent airway intervention by EMS providers. Among these, 13,999 were excluded for missing or invalid data on the timing of CPR initiation or advanced airway placement. An additional 5507 patients were excluded because ROSC occurred before or at the time of airway intervention. The final analytic cohort comprised 51,869 patients, with 27,591 classified as early AAM and 24,278 as delayed AAM. After TDPSM, 12,014 patients were included in each group (Figure 1).

### 3.2. Baseline Characteristics in the Entire Cohort

Among the 51,869 patients who received prehospital AAM, 27,591 were categorized as early AAM and 24,278 as delayed AAM.

The early AAM group had higher proportions of bystander CPR (69.1% vs. 56.1%, SMD = 0.272), initial shockable rhythm (15.8% vs. 11.7%, SMD = 0.108), and public arrest location (18.2% vs. 13.8%, SMD = 0.118). Supraglottic airway use was also more frequent (96.3% vs. 92.5%, SMD = 0.166). In contrast, IV line insertion (67.6% vs. 66.9%, SMD = 0.013) and epinephrine administration (24.7% vs. 22.4%, SMD = 0.056) were marginally higher in the delayed group.

The early group experienced shorter times to IV line insertion (5.0 vs. 8.0 min, SMD = 0.899) and epinephrine administration (8.0 vs. 10.0 min, SMD = 0.497). For in-hospital management, reperfusion therapy (7.0% vs. 4.7%, SMD = 0.097) and TTM (3.7% vs. 3.4%, SMD = 0.016) were more frequent in the early group (Table 1).

### 3.3. Baseline Characteristics in the Matched Cohort

Following TDPSM, the early (N = 12,014) and delayed (N = 12,014) AAM groups were well balanced across demographic, arrest, EMS, prehospital intervention, and hospital management domains.

Key variables included age (72.2 vs. 71.6 years, SMD = 0.038), female sex (36.7% in both groups, SMD = 0.003), diabetes (27.5% vs. 26.2%, SMD = 0.029), and initial shockable rhythm (16.4% in both groups, SMD < 0.001). In-hospital treatment variables were also balanced (Table 2).

### 3.4. Clinical Outcomes

In the matched cohort (N = 12,014 per group), early AAM was associated with superior clinical outcomes compared to delayed AAM (Table 3). The rate of prehospital ROSC was higher in the early group (11.8%) than in the delayed group (10.5%), with an adjusted RR of 1.21 (95% CI, 1.12–1.29). Favorable neurological recovery occurred in 5.8% of the early AAM group and 5.1% of the delayed group (adjusted RR, 1.12; 95% CI, 1.01–1.23). Adjusted models included confounders such as age, sex, medical history, urbanization level of residence, public arrest location, bystander CPR, multi-tier EMS response, response time, scene time, and type of advanced airway (Table 3).

### 3.5. SEM Analysis

SEM was conducted in the matched cohort to identify factors associated with time to advanced airway placement. The coefficients (b) represent unstandardized regression estimates, indicating the expected increase in airway placement time (minutes) per unit change in each variable. *p*-values are shown in parentheses.

Across all subgroups, both initial rhythm analysis delay and endotracheal intubation were consistently associated with longer airway placement times. In the group without epinephrine or defibrillation, significant predictors included initial rhythm analysis delay (b = 0.611, *p* < 0.05), multi-tier EMS response (b = 0.259, *p* < 0.05), and endotracheal intubation (b = 0.865, *p* < 0.05).

For patients receiving epinephrine, rhythm analysis delay (b = 0.515, *p* < 0.05), epinephrine administration delay (b = 0.244, *p* < 0.05), metropolitan location (b = 0.326, *p* < 0.05), and endotracheal intubation (b = 2.254, *p* < 0.05) were significant predictors. In the defibrillation group, rhythm analysis delay (b = 0.643, *p* < 0.05), defibrillation delay (b = 0.228, *p* < 0.05), and endotracheal intubation (b = 1.427, *p* < 0.05) were associated with increased airway placement time.

Among patients receiving both epinephrine and defibrillation, rhythm analysis delay (b = 0.539, *p* < 0.05), epinephrine administration delay (b = 0.202, *p* < 0.05), metropolitan location (b = 0.395, *p* < 0.05), and endotracheal intubation (b = 1.442, *p* < 0.05) remained statistically significant. Other variables, including public location, witnessed arrest, and bystander CPR, were not significant predictors in any subgroup (Table 4).

## 4. Discussion

This nationwide retrospective cohort study using TDPSM demonstrated that early AAM was statistically associated with improved neurological outcomes and higher rates of prehospital ROSC among patients with OHCA. These results suggest that earlier AAM may be linked to better outcomes, though this association should be interpreted within the broader context of prehospital care delivery and overall system performance.

Several mechanisms have been proposed to explain the potential benefits of early AAM during OHCA. Early airway placement may facilitate more rapid oxygenation and ventilation, thereby reducing hypoxia and hypercapnia during the critical early phases of resuscitation [20]. In particular, patients with a presumed respiratory cause of arrest may benefit from prompt airway management that restores effective ventilation [21]. Furthermore, earlier airway interventions may support uninterrupted high-quality CPR when performed efficiently and without interfering with other essential procedures [21]. It should be noted, however, that the observed associations may also reflect overall resuscitation quality rather than isolated effects of airway timing. Early AAM may serve as a marker of high-functioning teams that coordinate and execute all interventions efficiently. We explore this interpretation further through structural equation modeling below.

Despite theoretical advantages, the optimal timing and role of AAM during cardiac arrest remain uncertain. Prior studies examining AAM timing have yielded inconsistent findings, often influenced by population characteristics and study design. In in-hospital cardiac arrest, tracheal intubation during resuscitation has been linked to lower survival to discharge, possibly due to procedural delays and interruptions during critical interventions [22]. Conversely, studies of OHCA have reported mixed results. A secondary analysis of a randomized trial in North America identified no association between AAM timing and survival outcomes [23], whereas analyses of large Japanese registries found improved neurological outcomes when AAM was performed within 10 min in witnessed OHCA cases [7,8]. A Korean observational study reported no significant association between AAM timing and clinical outcomes, potentially attributable to unadjusted time bias and differences in EMS system characteristics [24]. Additionally, a regional study found that higher frequencies of prehospital AAM correlated with better neurological outcomes, underscoring the impact of EMS system performance over individual procedural timing [25]. Collectively, these findings indicate that early AAM may provide benefits in selected contexts, but its effectiveness likely depends on patient factors, EMS capabilities, and the broader resuscitation environment. Our SEM analysis suggests that AAM timing is closely linked to other critical interventions, indicating that early AAM may function both as a beneficial intervention itself and as a marker of overall resuscitation coordination. Disentangling these effects in observational research remains challenging.

To identify factors associated with airway management timing, SEM was conducted. Delays in initial rhythm analysis, epinephrine administration, and use of endotracheal intubation were each statistically associated with delayed AAM. Initial rhythm analysis, typically performed early in resuscitation, is unlikely to be affected by airway management. Thus, delays in rhythm analysis likely represent slower initiation of resuscitative actions overall. In contrast, epinephrine administration and airway management often proceed concurrently, suggesting that delays in one reflect broader inefficiencies in scene coordination and team functioning. The association with endotracheal intubation—a more technically demanding procedure than supraglottic airway placement—is consistent with previous research indicating that endotracheal intubation is associated with increased procedural time and complexity [26].

These results suggest that AAM timing should be interpreted within the context of coordinated resuscitation efforts rather than as an isolated procedural variable. While our primary analysis demonstrates an association between early AAM and improved outcomes, the SEM findings indicate this association may reflect both the direct benefits of timely airway management and its role as a component of high-quality, coordinated prehospital care. The observed correlation between AAM timing and other interventions does not negate the potential independent benefit of early airway placement, but rather contextualizes it within the broader resuscitation process. Delayed airway placement may not solely reflect a clinical choice to defer airway management, but may instead indicate broader issues with team dynamics, logistics, or procedural efficiency. Specifically, airway delay may represent a cascade in which delayed initial interventions and poor scene coordination impede timely execution of subsequent procedures such as vascular access and medication administration.

This study had several limitations. First, despite using TDPSM to minimize time-related bias and including broad covariates, residual confounding remains inherent to observational research. Key clinical variables including airway difficulty, anatomical factors, first-pass success rate, number of intubation attempts, and real-time patient physiological status at the time of airway decision were unavailable in the registry and may have influenced both the timing and outcomes of airway intervention. Additionally, the specific clinical rationale behind timing decisions (e.g., whether delay was due to clinical judgment, procedural difficulty, or circumstantial factors) could not be determined. Confounding by indication cannot be excluded, as patients requiring earlier intervention may have had distinct clinical trajectories affecting outcomes independent of timing. However, we attempted to minimize time-dependent confounding by setting CPR initiation as time zero and employing TDPSM to balance measurable covariates at comparable resuscitation time points. The absolute difference in favorable neurological recovery between groups was modest (0.7%), and given the potential for residual confounding, we cannot exclude the possibility that this difference reflects unmeasured differences between groups rather than a true treatment effect. Confirmation through prospective studies or randomized trials is needed.

Second, findings from this large, nationally representative South Korean EMS database may not generalize to other EMS systems. Endotracheal intubation comprised only a small proportion of cases, with supraglottic airways predominating, differing from systems in the United States or Japan where endotracheal intubation is more common and may affect outcomes differently.

Third, the study period encompassed COVID-19, during which respiratory arrests may have increased and EMS protocols changed. Pandemic-related impacts on airway management and outcomes were not assessed.

Fourth, TDPSM required dichotomizing patients using a 5 min cutoff based on median values for balanced matching, not established optimal timing. This pragmatic choice reflected the operational tempo of the Korean EMS system, where AAM is performed earlier than in other countries, making wider time windows used in prior studies incompatible with our data distribution. This prevented investigating whether outcomes vary incrementally with different timing thresholds, and optimal AAM timing remains undetermined and likely varies across EMS systems.

Finally, OHCA patients are clinically heterogeneous. Despite subgroup analyses, effect modification potential remains, as early AAM may benefit specific subgroups such as those with non-shockable rhythms or respiratory etiologies, warranting further study.

## 5. Conclusions

In this nationwide observational study of OHCA in South Korea, AAM within 5 min of CPR initiation was associated with higher rates of prehospital ROSC and favorable neurological recovery. SEM showed that airway delay was associated with delays in other critical interventions and with the use of endotracheal intubation, suggesting that airway timing is embedded within broader resuscitation dynamics rather than functioning as an isolated procedural decision. While these results suggest potential benefits of early AAM, the SEM analysis indicates that airway timing functions as both a component of and a marker for high-quality resuscitation. These findings are specific to the Korean EMS context, where supraglottic airways predominate and system characteristics may differ from other settings. Prospective studies and external validation in diverse EMS systems are needed to further clarify this relationship. Future research should evaluate whether system-level interventions to improve on-scene coordination and procedural sequencing can enhance resuscitation outcomes.

## Figures and Tables

**Table 1:** Patient characteristics of the entire cohort.

	Early Advanced Airway	Delayed Advanced Airway	SMD
	*n* = 27,591	*n* = 24,278	
Demographic characteristics			
Age, years	72.6	72	0.042
Female, *n* (%)	10,122 (36.7)	9146 (37.7)	0.02
Diabetes, *n* (%)	7374 (26.7)	6731 (27.7)	0.022
Hypertension, *n* (%)	10,691 (38.7)	9705 (40.0)	0.025
Stroke, *n* (%)	2539 (9.2)	2363 (9.7)	0.018
Heart disease, *n* (%)	5432 (19.7)	4831 (19.9)	0.005
Arrest characteristics			
Year of arrest			0.075
2019, *n* (%)	970 (3.5)	583 (2.4)	
2020, *n* (%)	8219 (29.8)	7716 (31.8)	
2021, *n* (%)	9117 (33.0)	7969 (32.8)	
2022, *n* (%)	9285 (33.7)	8010 (33.0)	
Urbanization level of arrest location			0.074
Metropolitan, *n* (%)	14,191 (51.4)	13,276 (54.7)	
Urban, *n* (%)	3694 (13.4)	3280 (13.5)	
Rural, *n* (%)	9706 (35.2)	7722 (31.8)	
Public place, *n* (%)	5010 (18.2)	3356 (13.8)	0.118
Witnessed, *n* (%)	11,342 (46.7)	12,258 (44.4)	0.046
Bystander CPR, *n* (%)	19,068 (69.1)	13,614 (56.1)	0.272
EMS characteristics			
Multi-tier response, n (%)	23,945 (86.8)	20,636 (85.0)	0.051
Response time, min [IQR]	9.0 [7.0, 12.0]	8.0 [6.0, 11.0]	0.155
Scene time, min [IQR]	14.0 [11.0, 17.0]	16.0 [13.0, 19.0]	0.453
Transport time, min [IQR]	8.0 [6.0, 11.0]	8.0 [6.0, 10.0]	0.062
Advanced airway management type			0.166
Supraglottic airway, *n* (%)	26,577 (96.3)	22,465 (92.5)	
Endotracheal intubation, *n* (%)	1014 (3.7)	1813 (7.5)	
IV line insertion, *n* (%)	18,472 (66.9)	16,403 (67.6)	0.013
Prehospital epinephrine, *n* (%)	6167 (22.4)	5998 (24.7)	0.056
Initial shockable rhythm, *n* (%)	4246 (15.8)	2837 (11.7)	0.108
Prehospital intervention characteristics (time to prehospital interventions from EMS-initiated CPR) [min]			
Advanced airway, median [IQR]	3.0 [2.0, 4.0]	7.0 [5.0, 9.0]	1.545
Rhythm analysis, median [IQR]	0.0 [0.0, 1.0]	1.0 [0.0, 1.0]	0.257
First defibrillation, median [IQR]	1.0 [1.0, 4.0]	2.0 [1.0, 5.0]	0.128
IV line insertion, median [IQR]	5.0 [3.0, 7.0]	8.0 [6.0, 10.0]	0.899
Epinephrine administration, median [IQR]	8.0 [6.0, 11.0]	10.0 [8.0, 13.0]	0.497
ED level			
Level 1, *n* (%)	6507 (23.6)	6270 (25.8)	0.108
Level 2, *n* (%)	12,620 (45.7)	11,727 (48.3)	
Level 3, *n* (%)	8464 (30.7)	6281 (25.9)	
Hospital management			
Reperfusion, *n* (%)	1930 (7.0)	1148 (4.7)	0.097
Targeted temperature management, *n* (%)	1026 (3.7)	832 (3.4)	0.016
ECMO, *n* (%)	344 (1.2)	310 (1.3)	0.003

SMD—standardized mean difference, CPR—cardiopulmonary resuscitation, EMS—emergency medical services, IV—intravenous, IQR—interquartile range, ED—emergency department, ECMO—extracorporeal membrane oxygenation.

**Table 2:** Matched cohort patient characteristics.

	Early Advanced Airway	Delayed Advanced Airway	SMD
	*n* = 12,014	*n* = 12,014	
Demographic characteristics			
Age, years	72.2	71.6	0.038
Female, *n* (%)	4414 (36.7)	4414 (36.7)	0.003
Diabetes, *n* (%)	3301 (27.5)	3145 (26.2)	0.029
Hypertension, *n* (%)	4627 (38.5)	4627 (38.5)	0.024
Stroke, *n* (%)	1124 (9.4)	1102 (9.2)	0.006
Heart disease, *n* (%)	2386 (19.9)	2386 (19.9)	0.012
Arrest characteristics			
Year of arrest			<0.001
2019, *n* (%)	356 (3.0)	356 (3.0)	
2020, *n* (%)	3998 (33.3)	3998 (33.3)	
2021, *n* (%)	4248 (35.4)	4248 (35.4)	
2022, *n* (%)	3412 (28.4)	3412 (28.4)	
Arrest location urbanization level			0.096
Metropolitan, *n* (%)	6015 (50.1)	6592 (54.9)	
Urban, *n* (%)	4352 (36.2)	3940 (32.8)	
Rural, *n* (%)	1647 (13.7)	1482 (12.3)	
Public location, *n* (%)	2461 (20.5)	1931 (16.1)	0.114
Witnessed arrest, *n* (%)	5451 (45.4)	5675 (47.2)	0.037
Bystander CPR, *n* (%)	8321 (69.3)	8321 (69.3)	<0.001
EMS characteristics			
Multi-tier response, *n* (%)	10166 (84.6)	10499 (87.4)	0.080
Response time, min [IQR]	8.0 [6.0, 11.0]	8.0 [6.0, 11.0]	0.015
Scene time, min [IQR]	14.0 [12.0, 17.0]	15.0 [12.0, 18.0]	0.184
Transport time, min [IQR]	7.0 [4.0, 11.0]	7.0 [5.0, 11.0]	0.006
Advanced airway type, *n* (%)			0.086
Supraglottic airway, *n* (%)	11,679 (96.9)	11,361 (95.2)	
Endotracheal intubation, *n* (%)	374 (3.1)	576 (4.8)	
IV-line insertion, *n* (%)	7637 (63.6)	8119 (67.6)	0.085
Prehospital epinephrine, *n* (%)	2236 (18.6)	2580 (21.5)	0.072
Initial shockable rhythm, *n* (%)	1968 (16.4)	2837 (16.4)	<0.001
Prehospital intervention characteristics (time from EMS-initiated CPR) [min]			
Advanced airway, median [IQR]	3.0 [2.0, 4.0]	8.0 [6.0, 9.0]	1.902
Rhythm analysis, median [IQR]	0.0 [0.0, 1.0]	1.0 [0.0, 1.0]	0.212
First defibrillation, median [IQR]	1.0 [1.0, 3.0]	2.0 [1.0, 4.0]	0.061
IV-line insertion, median * [IQR]	4.0 [3.0, 7.0]	6.0 [4.0, 9.0]	0.488
Epinephrine administration, median * [IQR]	8.0 [6.0, 11.0]	10.0 [7.0, 12.0]	0.280
ED level			0.113
Level 1, *n* (%)	2735 (22.8)	3053 (25.4)	
Level 2, *n* (%)	5443 (45.3)	5732 (47.7)	
Level 3, *n* (%)	3836 (31.9)	3229 (26.9)	
Hospital management			
Reperfusion, *n* (%)	906 (7.5)	906 (7.5)	<0.001
Targeted temperature management, *n* (%)	470 (3.9)	470 (3.9)	<0.001
ECMO, *n* (%)	155 (1.3)	194 (1.6)	0.027

SMD—standardized mean difference, CPR—cardiopulmonary resuscitation, EMS—emergency medical services, IV—intravenous, IQR—interquartile range, ED—emergency department, ECMO—extracorporeal membrane oxygenation. * IV-line insertion and epinephrine administration time variables were excluded from the TDPSM model because these procedures were not performed in all patients. Thus, SMDs for these variables do not represent covariate balance following matching.

**Table 3:** Survival outcomes by early advanced airway in the matched cohort.

	*n*/N (%)	Risk Ratio (95% CI)	
		Unadjusted	Adjusted *
Prehospital ROSC			
Delayed advanced airway	1258/12,014 (10.5%)	Reference	Reference
Early advanced airway	1414/12,014 (11.8%)	1.13 (1.05–1.21)	1.21 (1.12–1.29)
Survival to discharge			
Delayed advanced airway	929/12,014 (7.7%)	Reference	Reference
Early advanced airway	1016/12,014 (8.5%)	1.10 (1.01–1.20)	1.08 (1.00–1.17)
Good neurological recovery			
Delayed advanced airway	614/12,014 (5.1%)	Reference	Reference
Early advanced airway	699/12,014 (5.8%)	1.14 (1.03–1.27)	1.12 (1.01–1.23)

ROSC—return of spontaneous circulation, CI—confidence interval. * Adjusted for age, sex, diabetes, hypertension, stroke, heart disease, urbanization level, public location of arrest, witnessed arrest, bystander CPR, multi-tier response, response time, scene time, transport time, IV-line insertion, prehospital epinephrine administration, and initial shockable rhythm.

**Table 4:** Structural equation modeling of factors associated with time to advanced airway placement.

Variable	Advanced Airway Only (*n* = 16,239)	Advanced Airway + Epinephrine (*n* = 3859)	Advanced Airway + Defibrillation (*n* = 2979)	Advanced Airway + Epinephrine + Defibrillation (*n* = 957)
Initial rhythm analysis delay (min)	0.611 (<0.05)	0.515(<0.05)	0.643 (<0.05)	0.539 (<0.05)
Epinephrine administration delay (min)	-	0.244 (<0.05)	-	0.202 (<0.05)
Defibrillation delay (min)	-	-	0.228 (<0.05)	−0.039 (0.33)
Endotracheal intubation	0.865 (<0.05)	2.254 (<0.05)	1.427 (<0.05)	1.442 (<0.05)
Metropolitan	0.043 (0.33)	0.326 (<0.05)	0.045 (0.65)	0.395 (<0.05)
Public place	−0.03 (0.64)	−0.216 (0.08)	−0.003 (0.97)	−0.114 (0.52)
Multi-tier EMS response	0.259 (<0.05)	0.107 (0.60)	0.095 (0.49)	0.043(0.92)
Witnessed arrest	−0.023 (0.59)	−0.202 (0.13)	−0.064 (0.55)	−0.079 (0.67)
Bystander CPR	0.016 (0.73)	0.008 (0.94)	−0.112 (0.36)	0.156 (0.47)

EMS—emergency medical services, CPR—cardiopulmonary resuscitation. Values represent unstandardized regression coefficients, with *p*-values in parentheses. Time to airway placement (min) was defined as the interval from EMS chest compression initiation to advanced airway intervention.

## Data Availability

The datasets collected and analyzed in the current study are available from the corresponding author on reasonable request.

## Abbreviations

The following abbreviations are used in this manuscript:

OHCA	Out-of-hospital cardiac arrest
EMS	Emergency medical services
AAM	Advanced airway management
CPR	Cardiopulmonary resuscitation
TTM	Targeted temperature management
PCI	Percutaneous coronary intervention
ECMO	Extracorporeal membrane oxygenation
ED	Emergency department
ROSC	Return of spontaneous circulation
CPC	Cerebral performance category
CI	Confidence interval
RR	Risk ratio
TDPSM	Time-dependent propensity score matching
SEM	Structural equation modeling
IV	Intravenous
IQR	Interquartile range
IRB	Institutional Review Board
SMD	Standardized mean difference
AHA	American Heart Association
